# Alzheimer’s Disease and Its Possible Evolutionary Origin: Hypothesis

**DOI:** 10.3390/cells12121618

**Published:** 2023-06-13

**Authors:** James F. Whitfield, Kerry Rennie, Balu Chakravarthy

**Affiliations:** Human Health Therapeutics, National Research Council, Ottawa, ON K1A 0R6, Canada

**Keywords:** Alzheimer’s disease, evolution, hippocampal memory, entorhinal cortex, neocortex

## Abstract

The enormous, 2–3-million-year evolutionary expansion of hominin neocortices to the current enormity enabled humans to take over the planet. However, there appears to have been a glitch, and it occurred without a compensatory expansion of the entorhinal cortical (EC) gateway to the hippocampal memory-encoding system needed to manage the processing of the increasing volume of neocortical data converging on it. The resulting age-dependent connectopathic glitch was unnoticed by the early short-lived populations. It has now surfaced as Alzheimer’s disease (AD) in today’s long-lived populations. With advancing age, processing of the converging neocortical data by the neurons of the relatively small lateral entorhinal cortex (LEC) inflicts persistent strain and high energy costs on these cells. This may result in their hyper-release of harmless Aβ_1–42_ monomers into the interstitial fluid, where they seed the formation of toxic amyloid-β oligomers (AβOs) that initiate AD. At the core of connectopathic AD are the postsynaptic cellular prion protein (PrPC). Electrostatic binding of the negatively charged AβOs to the positively charged N-terminus of PrPC induces hyperphosphorylation of tau that destroys synapses. The spread of these accumulating AβOs from ground zero is supported by Aβ’s own production mediated by target cells’ Ca2+-sensing receptors (CaSRs). These data suggest that an early administration of a strongly positively charged, AβOs-interacting peptide or protein, plus an inhibitor of CaSR, might be an effective AD-arresting therapeutic combination.

## 1. Introduction

As the World population rose to 8 billion, there has been an increasing number of the super-aged with a disease we suspect has resulted from an evolutionary glitch resulting from the disproportionately immense neocortical expansion. It was Alois Alzheimer in 1901, while examining a behaviorally disturbed fairly young 51-year-old woman, who discovered that she had what we now know as the rare pre-senile or early onset version of this disease. After her death in 1906, he found that her brain was riddled with the now hallmark plaques and tangles. Then, it was Emil Kraepelin who, believing this to be a new disease, called it *Alzheimer’s disease* (AD) (*Compendium der Psychiatrie*, 1910) [1,2].

However, Alzheimer’s discovery was not as original as Kraepelin believed it to be. In fact, it was Oskar Fischer (1876–1942) who first saw the plaques (though not the tangles) in the brains of older senile patients with dementia [3]. Since tuberculosis was spreading throughout Europe at that time and since cerebral tuberculosis is accompanied by a slowly developing dementia with memory loss, he believed that the plaques were the tuberculosis-like Drṻsen (clubs) caused by the *Mycobacterium tuberculosis*-like *Streptothrix*. In fact, such infecting bacteria are carried to the medial temporal lobe via the middle cerebral artery, where they directly target the hippocampal memory-encoding machinery, like AD’s endogenous toxic AβOs (Aβ oligomers) discussed below [3]. Once the mycobacteria pass through the blood–brain barrier, they infect microglia, discard their cell walls, and produce mobile reservoirs of infectious bacteria. These bacteria can destroy the memory-encoding hippocampal machinery and spread upward along what appears to be an invariable AD trajectory from an entorhinal ‘ground zero’ to the neocortex [4]. Since Fischer was the first to describe the common late-onset form of AD, maybe we should call the amyloid deposits Alzheimer–Fischer or just Fischer plaques.

The common (>95 % of the cases) late-onset or sporadic (LOAD/SAD) version of AD starts stealthily in a late-middle-aged person who is unaware of the spreading destruction of networks, a *connectopathy*. This has likely started in the ancient memory-recording part of her/his brain that will clinically emerge only many years later. The disease is arguably triggered by relatively small numbers of AβOs, which are toxic soluble quasi-infectious oligomers of normally functioning monomers known as Aβ_x–42_s, and then driven and terminated by toxic hyper-phosphorylated tau oligomers (HPTOs). Here, we follow what appears to be the prescribed trajectory of this destructive Aβ_x–42_s → AβOs → HPTOs relay race through the brain [4,5] from the entorhinal cortex to the neocortex and finally the subcortical control panels and then suggest a novel way to potentially stop it.

## 2. Aβ Oligomerslikely AD-Starters, but Not the Terminators

AD starts from the failure of aging astrocytes’ and neurons’ control mechanisms to prevent their normally functioning Aβ_1–42_ monomers from over-accumulating and seeding cocktails of toxic AβOs. Such AβOs target and destroy synapses and, with them, networks and memories, spreading from the pathology’s ground zero to induce normal target cells to produce and release more of them. Thus, for example, injecting a tiny amount of an AβOs-rich AD brain extract into a healthy ‘humanized’ rodent’s hippocampus results in AβOs replication and induction of amyloidosis that spreads through the limbic region and beyond [6,7]. 

**Figure 1:**
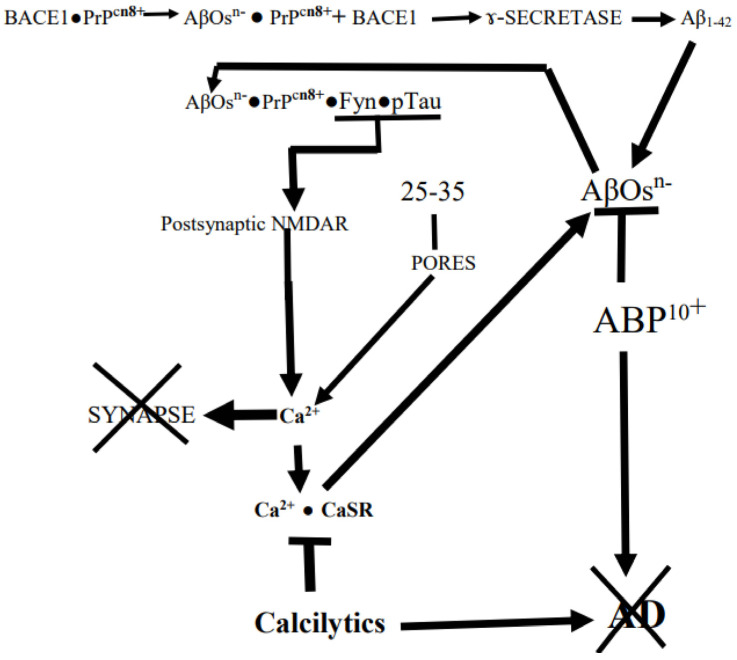
AβOs Selectively attack synapses. Schematic Representation of potential interactions of Aβ_1–42_ oligomers (AβOs) with various cellular proteins and modulating their functions leading to perpetuation of its own production and synaptic disruption in Alzheimer’s disease. As described in the text, negatively charged AβOs, upon binding to positively charged N-terminus of PrPc, releases the bound BACE1 and activate it. BACE1, along with γ-secretase, generates more AβOs-seeding Aβ_x–42_. AβOs, in collaboration with tau and Fyn kinase, also activate NMDARs, which increases intracellular Ca^2+^. AβOs can also increase intracellular Ca^2+^ by forming “membrane pores”. Together, they activate calcium-sensing receptor (CaSR), which can stimulate additional Aβ production to perpetuate the cycle. The cascade of such events can trigger synaptic degeneration leading to cognitive deficit in AD. Consequently, Aβ molecules, such as ABP, along with calcilytics that suppress CaSR activity, can potentially prevent the cascade of events that lead to Aβ-induced synaptic loss in AD.

In what follows, we will discuss how at the very core of AD is the destructive binding of AβOs to synaptic prions, PrP^C^s, riding on membrane lipid-rich rafts followed by synapse destruction, cognitive failure, and eventual neuronal death (Figure 1). This has been shown with mice which normally do not develop AD. However, when AD-susceptible transgenic mice carrying AβOs-producing human mutant APPswe/Psen 1∆E9 genes have their murine PrP^C^ genes knocked out, they can still accumulate AβOs. But now there is no loss of synaptic markers, impairment of memory, or early death: *the AβOs had lost their target* PrP^C^ [8]. Moreover, as we shall point out below (cf. reference [9]), “mutant” humans who cannot produce Aβs also cannot develop AD.

The interiors of neurons harboring such *potentially* dangerous proteins as Aβ_1–42_ monomers are turbulent nanocosms, loaded with dangerous IDPs (intrinsically disordered proteins) that are being continuously battered by the random Brownian thermal motion of water molecules that forces changes of their locations, conformations, interconnections, and interactions [10,11]. To functionally survive their inner maelstroms, neurons must keep their synaptic machinery intact and functioning [12]. For this, they have a ‘tool box’ containing potent, though age-sensitive, PQCs (protein quality and quantity control systems) that defend against protein conformation diseases such as AD. They do so by variously eliminating toxic oligomerized misfolded proteins, aggregated IDPs, and oxidatively damaged proteins using toxic agent-phagocytosing microglial cells, different types of folding catalysts, molecular chaperones and the potent protein-degrading proteasome [10,13,14,15,16]. 

So how and where are the AD-initiating AβOs made? Trumbore [17] has suggested that the principal region is outside of the cell. The brain has a cardiac pulsed interstitial fluid (ISF). Since active neurons secrete Aβ_1–42_ into the ISF [18,19], AD starts with hyper-accumulating, normally functioning Aβ_1–42_ IDPs being released from aging ‘Ground Zero’ entorhinal cells into the pulsing ISF [10,11,12,13,14,15,16,17,18,19,20,21,22]. It is the shearing action of the pulsing ISF percolating through the neuropil that produces strained Aβ^*^s, which, when sufficiently concentrated, collide with each other and thereby seed the toxic AβOs [17]. 

As the disease develops in its temporal–entorhinal cortical ‘ground-zero’ in a still cognitively normal but doomed aging brain, the specially structured and intra-neuronally situated Aβ_1–42_s content is rising. However, so far, there are no indications of the oncoming pathology, such as extracellular AβOs, plaques, or intracellular hyperphosphorylated tau tangles [22,23,24]. However, as we shall see, it appears that the expression of the CaSR (Ca^2+^-sensing receptor), an important contributor to the pathology around the dendritic spines and synapses, especially in the hippocampus, is increased by the rising Aβ_x–42_s monomers [25]. Moreover, because these are accumulating Aβ_x–42_s monomers’ ^14^HQKLVFFAEDVGSNK^28^ sequences resemble insulin’s ^19^BCGERGFFYTPKA^30^B sequence, when released into the ISF they will compete with insulin for its receptor and thus cause the early reduction of ^18^FDG (fluorodeoxy glucose) uptake observed in PET scans even before the significant appearance of the toxic AβOs which might be stronger insulin competitors [26,27,28]. These Aβ_x–42_s are more dangerously prone to misfold and, when released from the neurons seed the AβOs that destructively target the neurons’ spines and synapses. When with time, the increasingly accumulating, helix-coil Aβ monomers are released into the shearing ISF from the active synapses of such neurons, they can be distorted and aggregate into extracellular toxic ‘cocktails’ of the lethal AD-initiating AβOs, but some will later be *safely* ‘caged’ in, or attached to, the hallmark Alzheimer–Fischer plaques [29,30,31,32,33,34,35,36]. Larger aggregates in the pulsing ISF displace and distort synaptic complexes [34].

In addition, an important fraction of the Aβ_x–42_s accumulating in the AD brain have their N-terminal Aspartate (D) and Alanine (A) residues cleaved, leaving the newly formed N-terminal glutamate (E) residue. This is pyroglutaminylated into hypertoxic, AβOs-forming Aβp_E3-42_s by the increased level of pyroglutaminyl cyclase in the developing pathology [30,37,38,39,40,41,42,43,44]. Their importance for AD pathology is suggested by the paradoxical cognitive normalcy of some Aβ_x–42_s/plaques–loaded elderly brains’ failure to significantly make AβpE_3-42_ from the accumulating Aβ_1–42_s [39]. 

The physiologically functioning U-shaped Aβ_1–42_ monomer has a disordered, flexible anionic 1–17 N-‘tail’ and an 18–42 β-turn β-folded bilayer with a protease-resistant middle region. Within this region is the 24-28 bend and the C-terminal fold-stabilizing aspartate (D) 23- lysine (K) 28 salt bridge [10,45,46,47]. Four negatively charged D and E residues are concentrated in the unstructured N-‘tail’ (^1^DAEFRHDSGYE^11^). Because negative charges repel each other and reduce the hydrophobicity of the monomers’ C-fold ends, the E22s will tend to slow both the aggregation of monomers into soluble oligomers/protofibrils and their eventual collection into the insoluble fibrils/plaques by forming a stack of E22s [10,47]. Indeed, as expected, removing the negatively charged E22 residue (e.g., with the Osaka AD ΔE22, Arctic E22G, or Dutch E22Q mutations) does not eliminate the 24–28 bend, but by breaking the 22–28 linkage and loosening the pre-22 part of the fold *accelerates* oligomerization at least partly by increasing the hydrophobicities and eliminating the repulsion of the assembling monomers’ C-fold regions [10,30,46,48,49,50,51,52,53]. The Osaka ΔE22 mutation strongly increases the internal stability, which accelerates toxic oligomerization of Aβ_x–42_s in humans and “humanized” mice, but by reducing the flexibility of the mutant monomers, it impedes their insertion into fibrils and thus eliminates plaque formation in pulsing ISF [10,30,49,54,55].

According to this electrostatic model, large, soluble, negatively charged AβOs with their many flexible anionic 1-17 N-‘tails’ being buffeted in the ISF would be attracted to and bind to targets with strongly cationic (e.g., K^+^-rich) patches, especially those optimally configured for close contact with their targets. This indeed seems to apply to the PrP^C′^s (i.e., normal cellular prions with +vely charged N-terminus region) interaction at the core of AD’s synapse pruning [56,57,58,59,60]. Indeed, Kostylev et al. [58] have found that in “humanized” rodent models of AD brains, the PrP^C^s are targeted by a distinct population of soluble high molecular weight AβOs. In support of this it has been shown that PrP^C^s strongly bind to the high molecular weight AβO assemblies in AD patients’ brains but not to small synthetic AβOs [61]. As we shall see below, the principal feature of the disease is the AβOs → AβOs• PrP^C^s (cellular prions proteins, 57,58) → HPTOs chain of interactions that destroys synapses (Figure 1). The anionic AβOs trigger a multipronged cascade of events by binding to cationic PrP^C^s exposed to the ISF on cholesterol-rich lipid rafts in synaptic membranes where the PrP^C^s were restraining Aβ_x–42_s production by binding to BACE1, the β-amyloid precursor-cleaving enzyme 1 [62,63] (Figure 1). Thus, binding of AβOs to PrP^C^s initiates Aβ_x–42_s production cascade by dissociating PrP^C^s from BACE1, thereby activating the APP-cleaving enzyme [64,65]. The PrP^C^, itself an IDP, consists of a flexible N-terminal domain (residues 23–125) and a globular C-terminal domain (residues 126–254), which are anchored by the C terminus to the outer cell membrane leaflet via glycosyl–phosphatidyl–inositol. Specifically, the AβOs selectively bind to PrP^C^s [KKRPKGGGTHSQWNKPSKPKTNMK] cationic patch produced by the folding together of the 23–27 and 92–111 regions [42,65,66,67,68,69]. Interestingly, there is a negatively charged RNA aptamer that can induce the release of Aβ from AβOs• PrP^C^ complex by targeting PrP^C′^s two N-terminal positively charged patches [70]. Moreover, the normal physiological α-cleavage between residues 111 and 112 of PrP^C^ produces the N1^+^ (N-terminal 1) fragment that selectively binds AβOs and sequesters them in the extracellular space, which prevents them from accessing and destroying synapses [65,69]. Li et al. [71] have also shown that an antibody that selectively targets the N-tails, but not one that targets the C-termini, of soluble AβOs rescues the ability of the hippocampal CA1 cells to establish LTP in AD mice. As discussed below, there may be other cellular proteins such as pericentriolar material-1 (PCM1) protein and myristoylated alanine-rich C kinase substrate (MARCKS) protein that can potentially bind AβOs through their cationic patches, ^1276^KTFKTRKASAQASLASKDKTPKSKSKKRNSTQLKSRVKNI^1314^ and ^152^KKKKKRFSFKKSFKLSGFSFK^172^, respectively, and mediate their effects [72,73,74,75,76].

As we shall see further on, the cell surface, CaSR is another AD driver that induces its cells to produce and secrete endogenous AβOs-seeding Aβ_x–42_s when it is somehow selectively activated by exogenous AβOs [77,78] (Figure 1). When scanning the CaSR amino acid sequence for some kind of a cationic patch in this molecule’s bi-lobed extracellular VFT (Venus-Fly-Trap-like) domain, we found that there is indeed a small, strongly cationic R25-[Q26]-G27-K28-K29^3+^ patch at the tip of lobe 1 (Gorvin et al., [79]). But the CaSR is normally activated by cationic agents such as Ca^2+^ and polyamines such as spermine rather than the Aβ_1–42_ with its anionic N-tail or the polyanionic AβOs [78]. However, we suggest below that the CaSR-activation by AβOs is mediated by the Ca^2+^ surges resulting from the synaptic response to the AβOs → PrP^C^s interaction, which could account for the apparent selectivity of the activation of CaSR by AβOs (Figure 1). 

When AβOs bind to PrP^C^s in cholesterol-rich lipid rafts on synaptic membranes, they also trigger the principal destructive cascade that ends with a Fyn kinase-induced hyperactivation of neighboring NMDA receptors, producing an excitotoxic Ca^2+^ surge. However, if the AβOs are cleaved down to *chargeless 25–35 (GSNKGAIIGLM) peptides*, they will mimic the AβOs by simply binding to raft cholesterols and producing membrane pores through which excitotoxic Ca^2+^ surges occur [79,80,81,82] (Figure 1).

## 3. The Deadly AD Family

Histopathological studies have defined three sub-types of AD, (a) memory-impairing, (b) limbic cortex-attacking, and (c) hippocampus-sparing (thus memory-sparing) Alzheimer’s disease [83]. Here, we focus on the most studied hippocampus/memory-attacking AD.

The common (>95% of cases) LOAD/SAD is probably started by hyper-accumulating toxic AβOs-seeding Aβ_1–42_s in the ISF of a lateral entorhinal cortical nidus where the flow of primary data from various regions of the massive neocortex converge on the small entorhinal gateway to the hippocampal system. The pathology first spreads unnoticed from ground zero for decades along a limbic-neocortical trajectory, likely pruning synapses and inducing mature neurons to re-enter their cell cycle. This can potentially be detected in some people by REM sleep disturbances; by changing levels of Aβ_x–42_s and tau in the CSF (cerebrospinal fluid); by MRI and fMRI-demonstrable hyperactive yet shrinking hippocampi, swelling ventricles; by declining glucose uptake, and also, by the early appearance of phspho-tau ^181^in the bloodstream [27,84,85,86,87,88,89].

The incidence of LOAD/SAD increases exponentially in people living longer than 65 years [90,91], but the length of its asymptomatic onset varies from person to person. In addition to aging, the female gender, and several minor risk factors for LOAD/SAD, the only other major risk factor is having the ε4 allele of the Apo-e gene for the apolipoprotein-E (Apo E) protein. While ApoEε2 and ApoEε3 isoforms play major roles in lipid transport and injury repair, they do not increase AD susceptibility. However, heterozygous (i.e., ε3/ε4) or homozygous (i.e., ε4ε4) persons have twice or more the risk of developing LOAD/SAD [29,92,93,94]. A major reason for this is that ApoEε4 competes with Aβ_x–42_s for LRP1, which otherwise would bind the Aβ_x–42_s and rapidly carry them through the increasingly leaky aging BBB (blood–brain barrier) and release them into the blood [95]. It has also been reported that ApoEε4 impairs Aβs clearance by reducing the migration of human microglia-like cells and their phagocytic activities [96]. This reduced Aβ_x–42_s clearance is accompanied by increased Aβ_x–42_s production through increased AβPP processing [94]. The result of this is people with ApoEε4 carry a substantial amount of AβOs, which of course, increases the risk of developing AD. 

The very rare (~1% of AD cases) and faster developing AD is EOAD (early onset AD) or FAD (familial AD). EOAD/FAD symptomatically emerges as early as 40–50 years of age after a decade(s)-long unnoticed build-up, and it is thus less likely than LOAD/SAD to be distorted by any accompanying disease of aging.

Unlike the slow LOAD/SAD that emerges from its nidus and spreads along a trajectory of normal wild-type cells, EOD/FAD owes its much faster development to its post-nidal trajectory consisting entirely of mutant cells having three AβPP genes-carrying chromosome 21s (i.e., Down’s trisomy 21), *or* carrying a hyperactive mutant secretase such as BACE1(β-secretase 1) with its gene on chromosome 11), or presenilin 1 (γ-secretase 1 with its gene on chromosome 14), *or* presenilin 2 (γ-secretase 2 with its gene on chromosome 1), any one of which produces excessive amounts of AβOs-seeding Aβ_x–42_ monomers. These mutant cells also hyperproduce the Aβ_x–42_ monomers that are poised near the threshold of seeding the same ‘infectious’ toxic ‘cocktails’ of soluble oligomers that start LOAD/SAD [17,30,32,34,97,98,99,100,101,102,103,104]. 

The brain region on which we focus our brief glimpse of the ADs and their ‘infectious’ AβOs is the relatively small evolutionarily ancient EC (entorhinal cortex)-hippocampus complex connected to massive ensembles of neurons in the more recent immense neocortex.As we shall see, the brain uses this machinery together with the vmPFC/AC (medial prefrontal cortex/anterior cingulate cortex) to record, store, retrieve, and display objects in virtual form and more or less accurately replay events [105,106,107]. 

The human brain is an immensely complex electrochemical device consisting of billions of neurons in the cortex along with the same numbers or even more astrocytes communicating with each other via receptors, gap junctions, and nanotubes and, in the case of neurons, with various membrane potential oscillations from synaptic ensembles in dense clusters of rich-club cortical and subcortical core networks as well as sparsely interconnected peripheral networks [108,109,110,111]. Astrocytes literally cradle the neurons and collaborate with them via Ca^2+^ bursts (but not via action potentials) and gliotransmitters such as glutamate to form working ANTs (Astrocyte•Neuron Teams). Studies with Normal Adult Human cerebral Astrocytes (NAHAs) appear to suggest that the AβOs-driven intra-brain contagion can spread throughout the ANT networks via astrocyte gap junctions and tunneling nanotubes [112,113]. The immensity of this circuitry is indicated by the fact that just one astrocyte can contact 4–8 neuronal somata and enwrap as many as 140, 000 synapses in rat hippocampus, while a much larger human astrocyte can enwrap and communicate with as many as 2 million synapses [114,115,116,117]. 

A synapse is commonly regarded as a tripartite device consisting of a pre-synaptic and post-synaptic neuron around which is wrapped a process of a functional astrocyte collaborator [118]. There is a fourth component. If not soon overwritten by competing inputs, the synapse may be locked into a hole in a synaptic cradle which is a scaffold of CSPG (chondroitin sulfate proteoglycan)-associated ECM (extracellular matrix) [119,120,121]. By isolating and maintaining synapses, these ECM cradle holes provide storage sites for long-term memory consolidation and retrieval. However, the locking of synapses, and the spaces they occupy, in these ECM cradles increases with the age of the hippocampus and is associated with normal age-related plasticity and cognitive decline. Destroying the synapse-cradling ECM nets releases the synapses from their storage spaces and restores competitive erasure (plasticity) [119].

## 4. Neuron–Astrocyte collaboration

As we shall see further on, the neurocontagion’s toxic AβOs start AD by pruning synapses in the entorhinal cortex of the persisting ancient limbic core of the medial temporal lobe by being released into the local ISF from presynaptic vesicles and from EVs (extracellular vesicles, exosomes) produced by the autophagic intercellular signaling system [18,19,122]. Attracted by synaptic PrP^C^s, the infectious’ AβOs spread through the medial temporal perirhinal and parahippocampal gyruses, for example, into the neocortical rich-club networks of the posterior cingulate gyrus and parietal associational cortices, destroying synapses and disconnecting the circuits as they go [112,123,124,125,126,127,128,129,130].

As mentioned above, most synapses are enwrapped by the ANTs’ astrocytes. Armato and his colleagues have found that when exposed to Aβ_25–35_ (Figure 1), NAHAs produce and secrete both AβOs-seeding Aβ_x–42_s and HPTOs [77,113]. The AβOs that activate ANT astrocytes’ CaSR [121] stimulate them to produce various factors, one of which stimulates their neuron teammates to produce complement C1q, which tags post-synapses [131,132,133,134]. The C1q tag then induces the associated reactive astrocytes to additionally hyper-produce complement C3 which results in the postsynaptic deposition of AβOs•C1q•C3complexes. These complexes induce nearby-hovering microglia expressing the C3 receptor to phagocytose the tagged synapses [131,134,135,136]. In addition, AβOs also contribute to the synaptic destruction by triggering a destructive cascade on the PSD (postsynaptic density) by their selective binding to PrP^C^s N-tails (Figure 1).

The collaboration becomes dangerous when ANT astrocytes use their EAAT transporters to uptake spilled-over glutamate from their neuron teammate’s synaptic cleft in order to avoid destructive excitotoxicity. However, when the ANT neurons released Aβ_x–42_s start seeding AβOs in the ISF, they activate the partner astrocytes’ α-7nAChRs, the signals from which stimulate the astrocytes to release their accumulated glutamate [137]. This glutamate activates the neurons’ *extrasynaptic* NMDARs that trigger excitotoxic Ca^2+^ surges and events, including dysfunctional mitochondria pumping out ROS and destroying the synapse [137,138]. 

It is also likely that when ANTs’ neurons start making and secreting Aβ_x–42_s and seeding AβOs, it causes their astrocyte partners to do the same, but instead of dying, the astrocytes become sustained neuron killers and pathology spreaders by projecting the ‘contagious’ AβOs and their following HPTOs through the widespread astrocyte/neuronal network [126,139,140,141].

## 5. Where Does AD Start?

Our memory system began with the ancient medial pallium (Latin for *covering*) linked to olfactory lateral and proto-cortex dorsal pallia. In the various vertebrates, it has evolved over millions of years into a remarkably conserved *‘hippocampus wrapped in a unique neocortex’* [142,143]. The job of hippocampi extending from their pallial beginnings to now, is to rapidly record information from the neocortex into neuronal ensembles [144]. Small mammals such as mice can directly transmit primary neocortical information to the hippocampal system, but, as we shall see below, humans cannot directly transmit primary information to the hippocampal system from our massive neocortices [145]. Instead, we must transmit abstracted neocortical information to the hippocampal system.

The key feature of the evolution of primate brains has been the low growth of the limbic components (amygdala, entorhinal cortex, hippocampus, olfactory system, and septum) and the enormous growth of the human neocortex and that even includes it’s invading the brain stem and cord [146]. The current focus is on the likely pathological consequence of the enormously disproportionate expansion of the hominin neocortex, which began ~2.5 mya and ended ~3000 years ago [147]. This evolutionary neocortical ‘Big Bang’ happened without an equivalent expansion of the EC-hippocampus in the medial temporal cortex. This required the development of ways to manage the increased flow of messages converging on the memory system [130,148,149,150,151,152,153,154,155]. This challenge was met with an internet-like [156] increase in the routing of messages through the perirhinal and parahippocampal cortices via the EC to the hippocampus, which rapidly induces the cortical rubbing of the abstracted original event-inducing networks into interacting ensembles for on-cue replay [144,152,157,158,159].

These two cortical message collectors or abstracting routers are well described by Reagh and Ranganath [154], Michon et al. [148], Sekeres et al. [160], and Rudy [152]. Messages in the ventral stream carrying the gist of messages about objects from the perirhinal cortical neurons and in the dorsal stream carrying the gist of messages about spatial actions from the parahippocampal cortical neurons are delivered to the EC. EC then constructs a hexagonal neuronal grid to contain the hippocampal message-processing place cells and identify where the animal or human was when the messages were thus ‘*GPSed*’ by the EC-hippocampus system [143]. Cueing the combined activation of the originally induced group of cortical ensembles after they have been hubbed (wired) together in the neocortex by the integrating hippocampus gives a replay of the events—the what and where and when [143,161,162,163].

As we shall see below, a powerful device was invented about 2/3 of the way through the neocortical expansion, likely by *Homo erectus*, which channeled the enormous flow of information about the individual’s external and internal worlds from the neocortex to the temporal hippocampus memory recording system. This became the deeply embedded temporal library of relatively small neuronal ensembles, which evolved into the *words* of our current human languages. These words can be accessed, variously combined, and recorded by the hippocampus system to produce and store throughout the neocortex as sets of words, each of which, when expressed, activate the original huge ensembles of neurons that produce the images that make up the flow of consciousness. 

AD-like pathology emerges at low levels in the ECs of primates, such as aging macaque monkeys and very old chimpanzees, but it emerges far sooner and at a much greater level in the human LEC. AD is likely the product of the lifelong overloading of our memory-recording system, with immense amounts of data continuously streaming on it from the enormous neocortex that now occupies ~ 80% of the brain [164,165]. As pointed out above by Khan et al. [23] and Small and Swanson [166], AD starts in the tiny (only 1.3 × 10^5^/~1.6 × 10^10^ neocortical neurons; 0.3/1843 cm^2^ neocortical area) entorhinal-perirhinal border-zone (BAs 28b and 35). This region is uniquely structured to guzzle ATP and receive non-spatial messages from the ventral stream. The message is further routed through the perirhinal collector, along with the parahippocampal spatial messages from the dorsal stream, into the dentate gyrus-hippocampus and the vmPFC/ACC (medial prefrontal cortex/anterior cingulate cortex) system for ‘engramming’ [107,142,144,166,167,168,169,170,171,172]. 

Unlike any other part of the neocortex, the human LEC (Lateral Entorhinal Cortex) has bumps called *verrucae* (Latin for *warts*), which are visible to the naked eye. They contain dense clusters of large neurons with dendrites reaching up into the layer 1 bundle of axons carrying message packets from the perirhinal collector/router to the LEC gateway and from there to the dentate gyrus-hippocampus [24,166,173]. 

As pointed out above, the basic core of AD is the selective targeting and pruning of PrP^C^s-bearing synapses by AβOs. Therefore, this electrostatic attack by AβOs is probably most effectively carried out on neurons assembled, for example, to map exterior objects and events with their PrP^C^s-rich clusters of spines and synapses maintained in an LTP configuration, thus poised to replay the episode upon cue [152,174,175]. Each poised synapse’s post-synaptic component is loaded with increased numbers of actin filaments, clumps of PSD-95 associated with GluA2 AMPA receptors that, when activated by a Glu pulse, trigger a spike of Ca^2+^ by activated NMDARs The early reptilian proto-hippocampal medial pallium probably directly received and recorded the small amounts of minimally associated and edited primary sensory data projected into it from the tiny dorsal and the larger olfactory lateral pallia. As the evolving EC-hippocampal regions were induced to process ever-escalating amounts of diverse data from the expanding neocortex, they developed the two-collector system. The modern images in the modern conscious brain are produced by large interconnected ensembles of neocortical neurons. This probably did not challenge the ancestral, hippocampus-destined reptilian medial cortical pallium, which was as large or larger than the connected tiny dorsal cortical pallium and the then larger olfactory lateral cortical pallium [176,177,178,179,180,181,182]. The brains of our reptilian ancestors had no mammalian-strength neocortex. Their survival depended on such things as the OT (optical tegmentum) visual ‘Where’ system to locate and appropriately respond to predators, potential mates, and food sources. While on the other hand, the simple recognition of familiar salient objects depended on the olfactory and thalamic ‘What’ systems [183]. These things came together with the invention of the massive 6-layer, functionally diverse modules of the human neocortex wired together with long, invasive axons, which took control of the brain-stem OT and complexified the visual system. This created the four modern neocortical visual pathways, which included the OT ‘What’ pathway in the occiptotemporal pathway [183,184]. The medial pallium eventually became the hippocampal conformation, and its archaic lateral and dorsal pallial connections became, in part, the small LEC super-hub [178,181]. 

As mentioned in the Introduction, the AD’s AβOs are basically synapse-pruners and, therefore, network-disconnecting connectopathies. They are a kind of connectional diaschisis that spread along what appears to be a prescribed trajectory stretching from an EC nidus or ‘Ground Zero’ via parahippocampal gyrus to the retrosplenial cortex, posterior cingulate cortex, precuneus connector hubs, and the hub-rich default-mode network (DMN) [4,23,166,185,186,187,188]. Indeed, looking at the left medial hemisphere of the end-stage AD brain (Van Hoesen and Solodkin, Ref. [127]) and the striking overlap of the Aβ_x–42_s deposition with cortical hub sites described by Buckner et al. [187], one can trace the destructive trajectory of the pathology from its EC nidus in the medial temporal lobe to the synapse-loaded neocortical hub-way. According to the electrostatic model discussed above, the toxic AβOs spreading out of the EC are likely to follow hub-ways with synapses loaded with PrP^C^ targets in their PSDs.

So, after starting within the LEC nidus, AβOs likely spread upwards into the cerebral cortex leaving behind them a trail of highly visible amyloid plaques and pyramidal neurons stripped of their synapses [22,23,56,57,58,59,60,61,62,68,127,189,190,191,192]. However, AβOs seem to avoid the deeply anchored canonical sensory-motor regions (i.e., A1, MT, S1, and V1) and, at least initially, spare the basal ganglia-cerebellum non-declarative memory circuits [193]. 

## 6. Origins

The human brain, with its strikingly enormous neocortex dominating the relatively small limbic system, is the product of a disproportionate expansion of the neocortical prefrontal and the parietal association regions, with the vital memory-recoding machinery (including entorhinal cortices and the hippocampus) lagging behind [146,194,195,196]. This disproportional expansion of the neocortical/limbic region induced by the evolutionary neocortical ‘Big Bang’ might be a contributing factor in the development of AD in the longer-living, aging brain.

The current enormous 6-layer cortex is the product of a 3-layer reptilian-like brain consisting of periventricular sheets known as medial, dorsal, lateral, and ventral pallia, each with only one layer of pyramidal neurons [177,178,179,180,181,194,197,198]. Between the medial and lateral pallia was a narrow wedge of dorsal neuropil with an immense World-changing future—the enormous human neocortex. Something momentous happened in the third-layered (allocortical) brains of the mammal-like cynodontian reptiles that had survived the massive Permian Period extinction (~250 mya) and were on their way to full mamahood while coping with the emerging diurnal dinosaurs. These dinosaurs, with their special oxygen-conserving respiratory system, could thrive and grow in very low oxygen levels (~5–10%) during the ensuing Triassic and Jurassic Periods [199]. However, the evolving mammals, with their far less efficient respiratory system, had to stay small to cope with the oxygen lack and avoid the growing, evolving, and increasingly fierce dinosaurs. This forced them to shelter in burrows and function as much as possible in the cold at night with eye-supplementing-whiskers to scan and ‘feel-see’ things in dark places, advanced ear structures, and high-frequency communications to escape the attention of the ferocious diurnal dinosaurs [149,197,199,200]. 

One major brain-altering consequence of avoiding dinosaurs and coping with the lack of oxygen was a large expansion of the olfactory bulbs and the pre-piriform lateral pallium [146,181,194,201,202]. Consequently, the expanding lateral pallium slid over the dorsal pallial wedge to produce a potent double allocortical ‘sandwich’ with multiple layers of wide-ranging pyramidal neurons. This consisted of the overlapping part of the lateral cortex contributing layers II (2), III (3), and IV (4) and the underlying dorsal allocortex contributing the future layers V (5) and VI (6) of the unique mammalian neocortex. The non-overlapping, still three-layered part of the ancient lateral pallium stayed attached to the new neocortex as the allocortical piriform cortex [198]. 

Eventually, the mammals coming out of their nocturnal refuges took the first step on the road to the evolutionary ‘Big Bang’ and AD. With the extinction of the still-dominant and thriving non-avian dinosaurs as a consequence of the collision of a massive meteorite-asteroid with the earth, the early small mammals, with their novel neocortices, could spread out into the daylight and occupy terrestrial niches. The nocturnal limbic olfactory era then gave way to the diurnal audio/visual era. According to Paredes et al. [203], the increasing brain size and, with it, a lengthening RMS (rostral migratory stream) increasingly impeded and reduced the flow of progenitor neurons into the olfactory bulb. The lagging allocortical medial pallium stayed tightly connected to both the antique limbic olfactory region and the new elaborate neocortex and eventually became the hippocampus. Thus, was born the dangerously overstrained EC. 

Thanks to the deadly diurnal dinosaurs, our ancestors developed the 6-layer neocortex, consisting of a two-dimensional, ~2–3 mm-thick, ~2600 cm^2^ layer consisting of Mountcastle cortical columns (modules packed side-by-side and functioning according to the regions to which they are linked) [204,205,206,207]. Thus, was produced the strikingly gyrified (wrinkled) powerful human neocortex because this was the only way a neocortical sheet could enormously expand without avoiding conduction delays and supporting high synaptic connectivity.

The dentate gyrus•hippocampal memory-recording machinery in the brains of the rat-sized early mammals was nearly half the size of the overlying neocortex (which, as in the rat, is itself less than 15% of the entire brain). They were likely flattened banana-shaped allocortical tubes attached by their stems to the septal complex of thalamically and hypothalamically connected nuclei in the evolving temporal lobe [178,179,181,182,207,208,209,210,211]. Alongside the hippocampal slab was the amygdala, which was also attached by a short extension to another hypothalamus-connected septal nucleus, the bed nucleus of the stria terminalis [181,211]. With the growing temporal cortex pulling on septal connections, the hippocampal and the amygdalar short medial septal connections were circularly pulled down into the dentate gyrus’s indusium griseum, the hippocampus’s fornix and the amygdala’s stria terminalis [182,211,212,213]. Thus, was formed, the group of ‘cables’ stretching down over the striatum and thalamus to the amygdala, the dentate gyrus-hippocampus attached to what became the subiculum, parasubiculum, and, the temporal EC gateway along with the pyriform, perirhinal and parahippocampal collectors and routers of the LEC and MEC hubs from the diverse neocortical regions [178,181].

Between ~6 and ~2 mya, while a succession of African hominins (*Sahalanthropus*, *Ardipithecus*, and *Austalopithecus*) was progressively distancing themselves from the panin ancestor but holding the sizes of their brains at ~320–450 mL, they were drastically modifying their skeletons to become uniquely bipedal [214]. While the hominins (with 46 chromosomes) originally had chimpanzee-sized brains, their brain size ‘suddenly’ began growing as if destined for a 1000-pound super-gorilla. However, the mutating genes in bipedal chimpanzee-like hominins generated a neocortical ‘Big Bang’. The surge began with *Homo habilis*, who emerged ~2.5–3 mya with ~612 mL brains. Then, ~21 mya, on the way through the Big Bang came early *Homo erectus*, who had the first modern body form and a ~870-mL brain. Then, ~ 1 million-50,000 years ago came late *Homo erectus* with a ~ 950 mL brain. These growing brains were encased in extremely thick skulls with thick occipital tori and very thick supraorbital ridges [215,216]. The *H. erectus* brain was followed ~200,000 years ago by the massive ~1500 mL *H. neanderthalensis* brain and the ~1350 mL (~8.6 × 10^10^ neurons) 46 (23 pairs)-chromosome *H.sapiens* brain, *both of which* had disproportionately massive neocortices loaded with ~200 functional regions [180,195,197,216,217,218,219,220,221]. 

As mentioned above, the evolutionary expansion of our enormous neocortex and, with it, today’s AD began when the mammal-like cynodonts with their reptilian-type allocortical brains were forced to shift over to olfaction by the emerging diurnal dinosaurs. It now appears that another event leading to the enormous neocortical expansion happened ~14 mya when an ancestral primate’s cortical NOTCH2 gene duplicated into a functional and a pseudogene [222]. During this time, both the hominin and non-hominin primates’ neocortices were growing because of the expansion of the gestational cortical VZs (ventricular zones) and the formation and subdivision of the SVZs (subventricular zones) into inner and outer regions (iSVZs and oSVZs) with NOTCH2-promoted accumulation of the progenitor cells in the oSVZs [223,224,225,226]. 

The next event leading to the massive growth of the human and Neanderthal neocortices may have happened ~3–4 mya *only in a hominin ancestor* with PDE4DIP-NOTCH2NL by interacting with the NOTCH2 gene. The enormous growth of the hominin neocortex over the subsequent millennia triggered by these Notch-involved events was due to the truncated NOTCH2NL-Bs somehow increasing the level of NOTCH2 activity, particularly in the oSVZ of the developing hominin neocortex [227,228]. This NOTCH2NLs-induced NOTCH2 activity increased neocortical growth via the increased NOTCH2′s NICD-induced Hes1 gene activity that prolonged transit amplifying (TA) cell accumulation in the oSVZ [223,224,227,229].

Another contribution to the hominin neocortical expansion was made by the ARHGAP11A gene when its partial duplication included a single base substitution [230,231], which shifted the original ARHGAP 11A localization from nuclear importation into neural progenitor cells’ mitochondria [231]. This stimulated glutaminolysis which, like Hes 1 gene stimulation by NOTCH2, increased oSVC and upper neuron production by stimulating TA proliferation and increasing the number of cells to differentiate into neurons and, with this, an enlarged number of neocortical columns.

This massive expansion of the neocortical mantle with its huge cognitive leap forward from *Australopiths’* chimpanzee-sized brains to the expanding Homo brains was due mainly to increasing numbers and widths of the mini Mountcastle columns, enhanced prefrontal cortex’s executive functioning with increased axonal connections to the pre-motor and the parietal and temporal association regions [166,195,204,232]. The enormously increased cognitive power of the human neocortex also benefitted from cheaper, shorter, and denser interconnecting wiring by hemispherically lateralizing, cognitively advanced multimodal networks [233]. 

When the caudally increasing neocortex began pushing against the occipital cranial wall, it shifted its expansion downward and rostrally to produce a special primate protrusion, the temporal lobe of the memory machinery [181,206]. The pushing against the ventricular wall caused the allocortical plate to be forced into a sea horse (e.g *Hippocampus leria*)-like structure [181]. Although it was also growing, this ancient hub was only ~1.0–1.5% of the size of the massive neocortex. Though small, these relatively old complexes continued sending increasing amounts of data through the collector cortices into the EC-hippocampus for cortical hubbing of the event-participating networks [24,144,166,173,181,234]. This disproportionately expanding neocortex, now with more neurons than the other primate neocortices and an increased modal diversity of radial neuronal columnar units, resulted in the projection of enlarged streams of messenger packets to the LEC ‘hot spot’ gateway and through there to the hippocampus [166,181,197,234,235,236]. Thus evolved our powerful brain but with an age-hidden deadly glitch in the early short-lived humans. 

Despite its undersized LEC data nexus, the big brain served the short-lived (~20 years) populations extremely well because the common life-long youthful brains were protected by the anti-stress array of protective mechanisms. Then, the only AD in the small tribes of big-brained *Homo. neanderthalensis* and *Homo. sapiens* would have been very rare EOD/FAD mutants locally spreading the connectopathy. This could have been caused by inbreeding or, maybe, by funerary cannibalism as was practiced not very long ago by the Fore tribe of Papua New Guinea, as suggested by the death of a tribe member from the PrP^sc^- induced Creutzfeldt-Jakob prion encephalopathy they called Kuru [237]. However, now, in our long-living (~75 years) populations, there are increasing numbers of super-old people with brains having only declining PQCs and failing glymphatic disposal systems that cannot prevent LOAD/SAD. 

Finally, relatively short lives and lack of sufficiently disproportionately large neocortices could explain the very late emergence of an AD-like connectopathy in aging monkey and chimpanzee brains. Thus, for example, the human neocortex is ~3 times larger than the chimpanzee cortex, but it is without a correspondingly enlarged entorhinal cortex [217]. This human combination accelerates and magnifies AD emergence. However, when artificially infused into the lateral ventricles of the much smaller brains of aged female rhesus monkeys, human AβOs accumulate in layer 3 of their dorsolateral prefrontal cortices and in the hippocampi where, just as in human AD, they target PSD95 and destroy spines and synapses [238]. In other words, all that is required to start and accelerate the connectopathy in the primates is to provide an endogenous or exogenous source of AβOs, i.e., create an artificial, human-like overworking entorhinal gateway.

## 7. How Might AD Start?

Why are human LEC cells the AD starters? As mentioned above, the human LEC is structurally unique. Khan et al. [23] have found that the superficial layers of the AD-vulnerable LEC are extremely active; that is, they are a metabolic white-hot spot. The layer 2 cells are packed into striking bumps or verrucae, enmeshed in dense networks of blood vessels. Its neurons are loaded with mitochondria, and with the glucose and oxygen from the dense blood vessels, they generate ATP [239,240,241,242,243]. Obviously, this temporal region has evolved from overloading it with immense volumes of data to process from, for example, the conscious, awake neocortex. This is expensive; it requires lots of glucose and ATP. However, the production of toxic mitochondrial ROS byproducts (e.g., O^●−^ → H_2_O_2_) in the LEC verrucae [244] becomes especially dangerous for aging LEC neurons with their declining protective tool kits.

The EC FC and SC gateway cells’ function is to appropriately process and then project the abstracted cortical data from the perirhinal and parahippocampal collectors into the dentate gyrus and the hippocampus proper for hubbing cortical networks into a cueable engram to replay the cortical event. The ‘ground zero’ AD initiators are the hyperactive LEC fan cells. Because the LEC II (2) verrucal cells are so active, they have more AβPP and are thus prone to produce more Aβ_x–42_s than other neurons in normal brains. Thus, when their PQC systems start declining, they promptly start over- accumulating Aβ_x–42_s, and secreting them into the surrounding pulsing perivascular shearing ISFs, which energize them into AβOs-seeding Aβ^*^s [17,18,19,166,245]. In other words, the LEC data nexus is a medial temporal ‘hot spot’ that releases large amounts of Aβ_x–42_s into the pulsing ISF for making AβOs-seeding Aβ^*^s. As expected from this, Welikovitch et al. [22] have seen Aβ_x–42_s ominously increasing with age *in* EC neurons before the appearance of any AD hallmarks, even *in* post-mortem brains from *still cognitively normal individuals*. Also, there is a large decline of connectivity in the medial temporal lobe, probably because of the early onset of synapse pruning by AβOs before a significant decline in cognition [188,246].

Another feature of these dentate gyrally-projecting layer 2 cells is their reelin, homodimers of which are needed to produce hippocampal dendritic spines and synapses in the adult brain [247,248]. In aging brains, as the Aβ_x–42_s-clearing PQC systems are declining, layer II neurons start accumulating PrPCs, which bind to reelin and cause the assembly of non-functional reelin multimers [247,249]. Normally the reelin dimers activate the ApoER2 receptors that stimulate fyn to tyrosine (Y)-phosphorylate Dab1 adaptors and inhibit GSK3β [7]. However, unlike the reelin dimers, the AβOs-induced reelin multimers do not cause ApoER2-Dab1-mediated activation of the fyn pathway. Thus, this process activates GSK3β, which, in turn, phosphorylates tau and produces toxic HPTOs [249]. Thus, AβOs and HPT start their long connnectopathogenic process from the limbic region. 

Because the layer II (2) verrucae of the small LEC → hippocampal gateway are incessantly bombarded by multimodal messages from the huge neocortex, it becomes the most heavily damaged of all cortical regions by the AD connectopathy [241]. It costs a lot of ATP to process this data flow, but the EC ANTs must contend with toxic ROS-byproducts from their overworking synapses’ mitochondria [244,250,251,252,253]. This could be the reason why these neurons are so vulnerable to destruction. Indeed, the continual ROS-generating data processing in the hot spot is equivalent to focusing a destructive beam of ionizing radiation on it [254].

Neurons in the nidal EC layer 2 verrucae, like other active neurons, can make a lot of Aβ_x–42_s during SVC (synaptic vesicle cycling) and release it, through, for example, exosomes, along with the glutamate transmitter, into the synaptic cleft [18,19,29,166,245,247,255,256,257]. In the reelin-expressing EC ‘hot spot’ nexus of a young brain, these activity-generated Aβ_x–42_s are kept at a safe level by the cells’ diverse protection systems [16,18,19,29,255,256,257]. At this stage, the tau protein in the busy neurons is compartmentalized in the neuronal axons forming microtubule trackways in association with tubulin, along which the kinesin and dynein transporters carry cargos to and from the presynapses [258]. Tau is normally prevented from dangerously escaping into the somatodendritic compartment (SDC) by the AIS (axonal initial segment) filter [259]. As the brain ages, with the weakening PQC systems, neurons will start hyper-accumulating Aβ_x–42_s which seed AβOs that stimulate tau-hyperphosphorylating kinases such as GSK 3β. The hyper-phosphorylated tau detaches from axonal tubulin and can pass through the AβOs-impaired AIS into the SDC [260]. In addition, activation of fyn kinase signaling by the accumulating AβOs stimulates tau synthesis via MAPK (ERK), S6, and the loading of the SDC with hyperphosphorylated tau. Thus, the AβOs have set the stage for the massive multipronged network-destroying attack on the PrP^C^-displaying synapses [29,126,140,247,249,261,262,263]. 

Besides this, AβOs in the ISF also start the core synapse-pruning by selectively and avidly binding to PrP^C^ -displaying PSD to form a transmembrane signaling receptor complex with the mGluR5 (the metabotropic GluR5 receptor). This activates the SDC fyn kinase in PSD and contributes to synapse destruction along with the complement system-activated microglial cells, as described above. The activated fyn kinase can also hyper-activate neighboring ionotropic NMDAR, which destroys the synapse by triggering an excitotoxic Ca^2+^ surge through the receptor [29,68,264,265,266,267,268]. 

This is not all. As we outlined in more detail above, the AβOs also induce the ANTs astrocytes to induce the neurons to tag their synapses with complements of C1q and C3 to form C1q•C3 complexes that stimulate microglia hovering nearby in the ISF to phagocytose the synapse by activating their C3 receptors [131,132,133,134]. The AβOs also inhibit NKA-α3 (Na/K ATPase-α3), and with it, the ability to generate action potential and eventually open another way for toxic Ca^2+^ build-up [30,269]. 

As discussed above, the vulnerability of these energy-guzzling cells in LEC verrucae is also partly attributable to their synapses’ large loads of mitochondria that are also the targets of AβOs [270,271,272]. The AβO•PrP^C^•mGluR5•Fyn signaling complex converts the mitochondria from ATP producers to ROS producers and releasers of apoptogenic cytochrome c by disabling various mitochondrial targets, including the Complexes V and IV, and ATPsynthase [261,263,271,273,274,275,276]. Thus, these cascades of events stimulate the production of superoxide and its toxic products instead of driving ATP production, leading to the killing of cells and thus, destruction of the EC gateway [271,277]. The accompanying mislocalization of hyper-phosphorylated tau prevents any mitochondrial replacements from reaching the moribund neuronal synapses from the neuronal soma. [104,278]. Thus, the EC gates are closing, and the data/information-collecting olfactory, perirhinal and parahippocampal regions are disconnected from the dentate-hippocampus and, with this, episodic memory recording.

Until very recently, another likely participant in AD connectopathy has been ignored. Neurons, like most other mammalian cells, have immobile primary cilia bristling with various receptors and are key parts of the cognitive machinery [279,280]. They are most likely involved in AD because it has recently been shown that AβOs target the p75^NTR^ in the primary cilia of murine hippocampal neurons, the resulting signals from which impair recognition memory [281]. It is known that the AβOs in the ISF somehow collect at the ciliary base where they prevent such things as ciliary growth and p75^NTR^ and SHH [*S*onic *H*edge*h*og]signal transductions in the dentate-gyrus and thus contribute to spreading the connectopathy [280,281,282,283]. It is not clear how AβOs reach the ciliogenic machinery in the centrosomal hub. One possibility is that the AβOs in the pulsing ISF simply bind to the waving cilium and are carried down to the cilial base [17]. Another possibility might involve the PCM-1 protein. This is a 228.5 kDa protein that is believed to be involved in ciliary structure and function [284,285,286]. PCM-1 has a highly basic, K^+^-rich patch in its 1276–1314 region, which selectively binds AβOs [72]. Thus, we speculate that PCM-1 may pick up negatively charged AβOs with its polybasic patch and deliver them to the ciliary base and affect their function. 

## 8. The Spreading of LOAD/SAD Connectopathy

At the heart of the slowly spreading LOAD/SAD in the aging brain is the heavy intercellular traffic of EVs (extracellular vesicles, exosomes) along the main cerebral pathways [287,288]. The EV cargos are normally lipids, proteins, and mRNAs. The cargo might also include products such as the AβOs that are delivered by the attachment of the donor’s loaded vesicles to the recipients’ membranes, followed by the endocytic release of the AβOs [289,290]. The connectopathy is also locally spread from the nidus by the release of AβOs-seeding Aβ_x–42_s into the ISF by neuronal SVC and AβOs from periplaque halos [17]. 

As mentioned earlier, this happens at first asymptomatically and spreads from the nidus, with AβOs pruning synapses and disrupting the dense connections of the allocortical olfactory, amygdala complex, and the transitional entorhinal–allocortical hippocampal complex [7,23,35,104,112,126,127,188,262,291,292,293,294]. The spread of the connectopathy is likely maintained by at least two things: the selective attraction and binding of ISF AβOs to postsynaptic PrP^C^s, and the resulting Ca^2+^ surge-induced stimulation of CaSRs that induces the cell to make more AβOs [77,113,295]. 

When the ANTs in parts of aging brains, such as the hippocampus, start seeding toxic AβOs, they try to destroy them with their fading autophagic machinery. They inflate MVBs (multivesicular bodies) with the degradation-resistant AβOs that are eventually released as EVs into the ISF, with their toxic contents being delivered to nearby cells [7,29,287,288,289,290,296,297,298,299,300,301]. Moreover, instead of being released from neurons or astrocytes in EVs, some AβOs may enter the ANT cells’ nuclei and bind to the AβID (Aβ-interacting domain) regions of the AβPP and BACE1 gene promoters to increase endogenous Aβ production and enhance the spreading intra-brain infection [302,303].

In contrast, AD trajectory in an EOAD/FAD brain can spontaneously hyper-accumulate AβOs-seeding Aβ_x–42_s either because they have 3 chromosome 21s (Down’s syndrome), each carrying an AβPP gene or one of the two autosomal dominant secretase genes (e.g., presenilin 1). However, here too, the LEC is likely to be the nidus because these “mutant” neurons are also equipped to hyper-produce Aβ_x–42_ in their structurally unique EC nidal ‘hot spots’ although we would expect them to be maintained much closer to the picomolar ‘red line’ than pre-LOAD/SAD cells and likely to be pushed over it by earlier and therefore smaller declines in a younger brain’s Aβ_x–42_s clearance mechanisms [104,141,304,305]. 

## 9. CaSRs Participation in Driving the Connectopathy

Neuronal activity promotes the production and release of Aβ_x–42_s along with neurotransmitters from the ANT neurons, and thus the amount released into the pulsing ISF is a function of neuronal activity. Indeed, SVC is necessary for amyloidogenic AβPP processing [19,257,306]. In a normal plasma membrane, AβPP is compartmentalized into one set of small lipid rafts, and the individual secretases that cleave the Aβ_x–42_s out of it reside in separate rafts. This separation in the normal membrane is maintained by the MARCKS (myristolylated alanine-rich protein kinase C substrate) protein with its myristoylated N-terminus inserted into the membrane and its highly basic (K-rich) 152–172 patch bound to membrane PIP_2_s (phosphatidyl inositol bisphosphates). Under these conditions, AβPP is directly targeted by the non-amyloidogenic membrane α-secretase, ADAM 10, and produces a neurotrophic and neuroprotective sAβPPα fragment [257,307,308,309,310]. Besides synaptic stimulation, another early event in AD development appears to be increased PKC activity [311]. PKC phosphorylates several sites on MARCKS protein (S159, S163, S167, S170), which causes the strongly positive K-rich 152–172 patch to become less positive and thus separate it from PIP_2_. This permits the fusion of lipid rafts, which allows the interaction of AβPP with secretases resulting in the production of Aβ_x–42_s [77,257,307,308,309,310,311,312]. When the neuron empties these loaded synaptic vesicles, the Aβ_x–42_s are released into the synaptic cleft along with the neurotransmitter [22]. The released Aβ_x–42_s in the pulsing ISF then seeds AβOs that can infect more distant cells as described earlier [17,122,257,287,288,296].

A consequence of the Aβ_x–42_s accumulation in the aging EC cells appears to be the stimulation of CaSR expression [25]. Small locally produced AβOs-‘barrels’ lined with their negatively charged N-‘tails’ are inserted into the cell membranes and enable a Ca^2+^influx, which would activate the CaSRs [10,17,30,77,78,257,313,314,315]. The AβOs can also stimulate CaSR via the AβOs → PrP^C^s → mGluR5 → NMDA triggered Ca^2+^ surge described above. Most importantly, activated CaSRs are also AβOs replenishers that maintain the ‘contagion’. Thus, there are two ways the anionic AβOs can selectively induce connectopathy-driving reactions, one via Ca^2+^•CaSR-mediated production and release of AβOs seeding Aβ_x–42_s, and another by reducing ADAM10 and increasing AβPP [77,78]. Moreover, the AβOs → Ca^2+^ → Ca^2+^•CaSR signaling induces various harmful cytokines [77,78,112,113].

## 10. Why Is LOAD/SAD a Disease of Aging?

As pointed out above, the densely crowded and intricately structured cellular inner nanoworld, be it neuronal or astrocytic, is constantly battered by the aqueous Brownian maelstrom [12,14]. Thus, the complex interacting nano-devices in such a place must constantly be repaired or replaced. As these systems decline over the years in the wild-type brain, the damage mounts, and LOAD/SAD is one of its many consequences. An example of one such important decline in the aging brain is that of BDNF, likely because of the reduced physical exercise of older people [316]. 

The high activity forced on the neurons in the EC gateway-hippocampus complex in a young brain produces relatively large amounts of Aβ_x–42_s which normally promote synaptic plasticity and episodic memory recording provided they are kept at or below picomolar levels [18,29,245,247,255,256]. As the PQCs decline with age, some of the increasingly uncleared Aβ_x–42_s seed mixed ‘cocktails’ of toxic AβOs in the ISF and on the surfaces of large plaques [17,34,77,98,269,278,317,318,319]. In the healthy young brain, PQCs prevent Aβ_x–42_s surging above the physiologically safe picomolar level. For example, Aβ_x–42_s/AβOs can be cleared by zinc-metalloproteinases such as insulysin and neprilysin and/or by transportation across the blood–brain barrier (BBB) into the blood circulation by lipoprotein receptor-related protein-1 (LRP1) [98,320,321,322]. Then, there is also the glymphatic system in which networking astrocytes take up waste from the brain with their neurovascular endfeet and drain it into the peripheral lymphatic circulation [17,323,324,325,326,327]. 

Glymphatic processing starts in CSF from the four ventricular choroid plexuses flowing out of the fourth ventricle into the SAS (subarachnoid space) through the foramina of Magendie and Luschka [328]. As the CSF flows through the SAS, portions are pulled down into the perivascular Virchow–Robin spaces by the pulsing arteries and arterioles. Stationed along these pulsing vessels are phalanxes of astrocytes attached to them by end-feet containing AQP4 (Aquaporin 4) water channels [327,328,329,330]. This astrocyte system, functioning optimally in a young sleeping brain, sends a bulk flow of waste-bearing ISF through the large veins, into the arachnoid granulations, the superior sagittal sinus, and finally into the peripheral circulation. With advancing age, the penetrating arterial and arteriolar walls stiffen, and thus, the glymphatic system’s principal pumps weaken, the phalanxes of periarterial astrocytes disperse, and the Aβ_x–42_s/AβOs-bearing ISF flow slows [326,327,331]. Along with this, the system is likely being progressively dismantled with the perivascular AQP4-bearing astrocytes being dispersed by the accumulating AβOs. Various cytokines, as well as NO and its toxic derivatives and MMP9 (Matrix MetalloProteinase 9), produced by astrocytes in response to AβOs, disrupt the claudin-attached BBB lining of the blood vessels [327,330,332]. The destructive impact of the declining sewage system on the cognitive machinery is increased by the build-up of high molecular weight AβOs not being clearable from the interstitial fluid [30,333].

## 11. The Clinical Emergence of LOAD/SAD after Its Long Stealthy Prelude

As the connectopathy spreads outwards and upwards from its shrinking nidus, it leaves a trail of harmless Aβ_x–42_s monomers and toxic ‘cocktails’ of ‘infectious’ AβOs that will destroy, for example, the hippocampus, the posterior cingulate gyrus, and parietal cortical rich-club networks, but not primary motor, or somatosensory areas [127,128,321,334,335,336,337,338]. However, the Aβ_x–42_s and AβOs from the cells are locked into the dense cores of large senile plaques that prevent them from spreading the contagion beyond their immediate neighborhoods [34]. However, Aβ_x–42_s being squeezed and stretched by flowing over the surfaces of large plaques can seed AβOs that can attack and prune nearby synapses [34]. Most importantly, the plaques can mark the trajectory of the connectopathy from the EC to its neocortical targets [339,340,341]. The changes in the levels of the Aβs in the blood and CSF also reflect the kinetics of formation and clearance of Aβ_x–42_s/AβOs and have enabled the detection of the spreading pathology much before the onset of clinical symptoms [27,34,342,343].

Along with synaptic pruning and cell cycle initiation, there is a surprising pre-plaque fMRI-detected surge of false hyperactivity in the hippocampus despite the degenerating fornix and the EC layer II2 [23,86,87,133,317,344,345,346,347,348,349,350,351]. Despite this spurious hyperactivity, there is a significantly impaired functioning of the dentate gyral/CA3 regions in MCI brains, along with their shrinking hippocampi [352,353]. 

One cause of this early hippocampal hyperactivity could be the accumulating AβOs that activate CaSRs that can hyperactivate hippocampal pyramidal cells by downregulating their GABA-B-R1 receptors [113,354,355,356]. A related reason could be the increased MMP-9 levels that damage the pyramidal neuron-restraining hippocampal GABA-ergic PV+ (parvalbumin) interneurons by destroying their protective PNNs (perineuronal nets) [357,358]. 

Since fMRI gives a signal based on blood oxygen and volume levels [359], another possible contributor is the AβOs-stimulated release of VEGF (vascular endothelial growth factor) and proinflammatory cytokines (i.e., Il-1β, IFN-γ, TNFα) from astrocytes’ end feet attached to hippocampal blood vessels during the prolonged presymptomatic period. Such a sustained VEGF bombardment should drive angiogenesis and increase the EC gyral/hippocampal microvasculature density and, with it, the BOLD fMRI signaling responses [354,360,361,362,363,364,365,366,367,368]. An enhanced surge of blood through such an expanded vasculature would flood the shrinking hippocampus with oxygen and glucose, thus instead of a fading BOLD fMRI signaling, there is supernormal BOLD signaling peaking in mid-MCI [86,353,368,369,370,371]. This means that a hypersurging blood flow along with damage to the GABAergic PV+ neurons will make DG/CA3 cells hyperoxic, hyperglycemic, hyperactive, and, consequently, hypofunctional [364]. Moreover, and most importantly, the hyperactive neurons will produce and release into the ISF increasing amounts of AβOs-seeding Aβ_x–42_s [19,86,344,372,373]. 

The post-MCI collapse of the BOLD fMRI signal when the brain converts to full-blown AD is likely, at least in part, due to the sustained spreading of perforation and severing of blood vessels, particularly in the hippocampus induced by Aβ (Aβ_1-40_) deposits and the production of NO and its toxic derivatives [354,362,366,374,375]. There will be spreading regions of hypoxia from the vascular damage leading to a buildup of HIF-1 (hypoxia-inducible factor-1), which in turn would stimulate Aβ_x–42_s/AβOs production by activating BACE1 and γ-secretases [360,376,377,378,379,380]. Along with this spreading BBB breakdown are the local leakages of toxic serum components into the brain, which can activate astrocytes and microglia to produce inflammatory cytokines. This is accompanied by a spreading shortage of glucose and thus ATP [327,381,382,383,384,385]. Thus forms the basis of the hypo-metabolism indicated by declining ^18^FDG-PET signaling in regions along the AβOs’ trajectory, such as the PCC and precuneus [27].

As conversion to full-blown AD nears, not enough damage has so far been done to disrupt daily activities, but the roiling intra-brain pathological activity could be seen in disappearing EC layer 2 verrucae, shrinking hippocampi, and cerebral ventricles swelling [27,241,242,251,382,386,387,388,389]. However, the spreading of AD connectopathy becomes increasingly entangled with other pathologies-of-age, especially neurodegeneration-promoting cardiovascular diseases. Eventually, the spreading damage reaches the threshold of irreversibility. 

## 12. The Lethal Tau-Driven Finale

In the EC of an aging but still cognitively normal person, there is a harmless progressive accumulation of Aβ_x–42_s with no AβOs. Eventually, the accumulating Aβ_x–42_s reach the toxic AβOs-seeding level and the onset of AD connectopathy as indicated by the generation of Ptau-T181 and its release into the circulation [88]. AD is actually a terminal tauopathy in which the currently invisible toxic AβOs start the connectopathy by inducing the mislocation of normal tau from the axon to the SDC by stimulating its hyperphosphorylation. This produces the unfolded toxic tau that collaborates with PrP^C^s to destroy synapses [260,390,391,392]. So, the hyperaccumulating AβOs produce the toxic HPTOs that actually kill the cells and, like AβOs, are also able to prion-like spread the clinical symptoms [5,22,126,390,393]. Thus, the developing pathology has entered the final stage with the toxic AβOs-induced HPT/HPTOs’ spreading, destroying synapses, disconnecting circuits, and filling neurons with the hallmark NFTs (neurofibrillary tangles) [292,394]. The person converts into full-blown dementia, in which he/she may survive semi-functionally, but not at all cognitively, for a few more years [5,141,262,293,395,396,397,398]. 

According to Ittner and Götz’s delightful metaphor [140], Aβs and taus are parts of a toxic two-step (*pas de deux*) choreography. To paraphrase Bloom [139], AβOs load the AD gun with HPT ‘bullets’ and then fire it to kill the cell. Consistent with this, it has been reported that in triple transgenic mice expressing *both* human Aβ_x–42_s and the mutant human tau P301S, primary cilia in dentate granule cells were significantly shortened (~50%), but not in transgenic mice expressing only human Aβ_x–42_s [280,282]. Chiarini et al. [304] have reported that NAHAs, treated with Aβ_25–35_, overproduce HPT, package it into exosomes, and release them into the culture medium. Rapoport et al. [399] have reported that neurons expressing either human or mouse tau degenerated in the presence of Aβs, but tau-depleted neurons were unaffected. Roberson et al. [400] have reported that reducing endogenous tau in AD-model mice reduced Aβ-induced actions. Similarly, Tackenberg and Brandt [401] have reported that Aβ_x–42_s alone were *not* toxic for cultured transgenic murine hippocampal CA3 neurons, but there was a massive neuronal degeneration in these Aβ-treated cultures when tau was also expressed and made toxic by hyperphosphorylation and oligomerization to HPTOs. Khan et al. [23] have further illustrated this by showing that exposing mice to either a mutant human AβPP or a mutant human tau did not significantly affect them. Expressing them together damaged the mice. More recently D’Avanzo et al. [395], Jorfi et al. [402], and Takeda et al. [403] have shown that 3-D cultures of human neural progenitor cells produced with iPSC (induced pluripotent stem cells) derived from AD patients accumulated Aβ_x–42_s and human HPTOs, but selectively decreasing Aβ_x–42_s production with BACE1 or γ-secretase inhibitors decreased HPTOs. Finally, an elderly woman in Colombia was carrying her family’s presenilin 1 gene and thus was expected to develop AD early in life like all of her relatives [404]. As expected, her brain was loaded with plaques, but unexpectedly she had no tau tangles in her brain, and she had somehow escaped AD’s hyperphosphorylated tau [404].

## 13. The Synapses Pruning AβOs-PrP^C^-Tau Combination Also Induces Neurons to Suicidally Try to Enter Their Cell Cycle

It appears that something surprising and important starts with the asymptomatic onset of the AβOs-triggered AD that may be responsible for as much as 90% of the eventual neuronal death [405,406]. The aging neurons, with their declining nano-machinery trying to survive the toxic onslaught of AβOs in the EC and the hippocampus, unsuccessfully attempt to switch on their cell survival program that includes cell proliferation [190,405,407,408,409,410,411,412]. This is surprising because such mature neurons should have permanently dismantled their cell cycle machinery. This attempt to proliferate could be due to AβOs inducing the synapse-pruning microglial cells as well as astrocytes to produce enough IL-1, IFN-γ, TNFα, and VEGF to stimulate the neurons to attempt to restart cycling in the hippocampal CA1 and CA3 regions [112,405,406,407,408,411,413,414,415]. However, according to Kodis [190], the initiation of cell cycling is likely due to the AβOs-induced phosphorylation of tau at the Y18 residue, which activates fyn kinase in dendrites and spines. Activated fyn kinase phosphorylates NMDA receptors, which cause a Ca^2+^ influx that triggers cell cycle re-entry [416]. 

The cell cycle suppression in normal mature neurons is partly due to the confinement of DNA-replication-driving genes in dense chromatin along with a high level of Rb (retinoblastoma) protein that blocks the expressions of these genes by blocking their contact with the E2F transcription factors [417,418]. This proliferative silence is broken when the AβOs induce chromatin-restructuring and upregulation of miR-26b that derepresses the set of E2F-responsive genes such as the replication-initiating Cdk2-cyclin E. [408,418]. The AβOs, also stimulate the MEK-ERK pathway that turns off TAp73 and, with it, the miR-34a that has been blocking the production of the cell cycle-initiating cyclin D1 kinase [415]. 

These activities start the buildup of chromosome replication. Some neurons can replicate their DNA and become tetraploids, but more likely, they become genetically unbalanced aneuploids by only partly replicating their chromosomes [419]. However, none of the neurons can initiate prophase because, in the AD brain, Cdk1•Cyclin B1 remains in the cytoplasm associated with HPT and NFTs and cannot get into the nucleus to trigger the events that break down the envelope [406,407,408,420,421]. The danger of this is that Cdk1•Cyclin B1 marooned in the SDC stimulates kinases such as Cdk5 and GSK-3β to further load the SDC with HPTOs [139,397]. If these are not challenging enough, the cytoplasmic Cdk1•Cyclin B1 also phosphorylates and activates the apoptogenic Bad, which, if not blocked by BDNF (which is reduced in aging brains [316], would induce an aneuploid pyramidal neuron to suicidally start the destructive restructuring needed to enter mitosis [407,408].

## 14. Do AβOs Really Co-Drive AD Pathology?

There are doubts about AD being triggered by Aβ and thus questioning the amyloid hypothesis of AD [422]. One of the main reasons for this is that several anti- Aβ antibody-based therapeutics have failed in clinical studies. Because of the wildly held incorrect belief that AD is caused by the plaques, it was felt that it should be treatable by plaques-eliminating agents [423,424]. This has recently been tested, for example, by Biogen/Eisai’s monoclonal anti-Aβantibodies, aducanumab (ADUHELM, [425]), and lecanemab (Leqembi, [426]). While they did indeed significantly reduceAβ-plaques, *they only marginally reduced the patient’s cognitive decline.*

However, Dodart et al. [427] reported that a monoclonal anti-Aβ antibody did rapidly reverse memory impairment but *without reducing Aβ deposition* in the cortices or hippocampi of PDAPP AD Tg mice. Chui et al. [428] reported that transgenic mice carrying the human presenilin 1 gene suffered cognitive decline also without Aβ plaques. AD patients carrying the E22-less Osaka ΔE22 mutant are severely cognitively impaired with AβOs in their CSFs, but extremely low levels of plaques [28]. Knight et al. [429] have reported that AβOs, which accumulated in the brains of mice carrying the Osaka ΔE22 mutant, were associated with memory defects again without plaques. Lesné et al. [388] have reported that injecting purified AβOs into the ventricles of wild-type rats dramatically reduced spatial memory formation. In other words, the histologically striking plaques or deposits do not correlate to the brain damage in rodents and humans, while it is increasingly evident that the histologically invisible small amounts of diffusible high molecular weight AβOs, do correlate with brain damage with or without accompanying Alzheimer-Fischer plaques. Finally, the most convincing role of Aβ in AD development is the inability of a group of Icelandic people with an impaired amyloid APP gene to develop the disease [9]. 

Thus, the AD treatment failures are likely due to using a wrong model of this connectopathy. It is initiated by AβOs instead of the very visible later-appearing Alzheimer–Fischer plaques. It first irreversibly advances unnoticed for decades in aging EC neurons and then extracellularly along its characteristic trajectory from the limbic origin to the neocortex, irreversibly pruning the synapses. However, in some people, the consequences of the stealthy AβOs-inflicted EC-hippocampus damage develops a decade before clinical AD. As outlined above, the multipronged attack on synapses, particularly those of pyramidal neurons, by AβOs•PrP^C^ complexes also include the production of toxic HPTOs by Fyn and Pyk2. Unfortunately, the progression and staging of the pathology in humans have been based too late on accumulating plaques and tau tangles instead of the onset of AβOs production, which would be the best time to start treatment. This problem may be on the verge of the solution by the ability to detect the early AD-specific appearance of P-T181 [88] or the recently reported brain-specific tau [89] in blood plasma. Unless the pruning is stopped *before becoming functionally noticeable in an MCI patient*, the AβOs-induced HPTOs might even have independently started driving a secondary tauopathy. However, just reducing Aβ_x–42_s and the pertinent AβOs then can have little or no effect on cognitive decline. A combined anti-AβOs and anti-HPTOs treatment would have to be combined to control this. Even if this were to be initially effective in preventing the accumulation of AβOs, it would have to be continued because of the aging brain’s unfortunately unstoppable ability to continuously generate AβOs. 

In summary, there are two major reasons for the current failures of the clinical trials of putative Aβ -based AD therapeutics. The first is not knowing which of the heterogeneous AβOs in the toxic cocktails triggers the AD pathology. Towards this end, recent clinical data on the Aβ-protofibril-targeting antibody therapeutic, donanemab, may shed some light on this [430]. The second is we do not yet know how to detect the asymptomatic onset before the appearance of PETscan-detected plaques to prevent the pathology from starting. Obviously, it would be ideal to be able to give *routin*e (e.g., yearly) MRI brain scans to discover and follow the development of early asymptomatic hippocampal and ventricular structural changes. However, this has been impossible because of the very high cost. The invention of a small, very much cheaper, mobile (on wheels), radiofrequency-free Ultra-low-field MRI scanner may be helpful in addressing this issue [431].

## 15. How Might AD Be Treated?

The principal features of the LOAD/SAD connectopathy are: it starts in the LEC of aging brains, and it does so by asymptomatically spreading, slowly disrupting cognitive circuitry, one or two decades before patients, families, and physicians are aware of anything being amiss. Clearly, the most effective way to treat the developing pathology would be to strike it directly on ‘Ground Zero’ as soon as possible. However, before considering treatment options, we must still find a way to detect AD onset long before the accumulation of Alzheimer–Fischer plaques and tangles. Perhaps something like the appearance in the bloodstream of molecules such as T181-p-tau [88] or the recently described brain-specific tau [89] that are easily measurable might be helpful, in addition to other early biomarkers.

From this brief overview of some of the growing number of AβOs’ targets in LOAD/SAD brains, it appears that the core of AD connectopathy advancing through the brain is AβOs → PrP^C^s → Ca^2+^•CaSR → HPTO (Figure 1). This destroys synapses and induces mature neurons to suicidally try re-entering their cell cycles. It follows that the connectopathic cascade might be stopped by an avid AβOs binder, such as monoclonal antibodies or basic peptides [72,73], which would prevent AβOs from triggering the cascade by binding synaptic PrP^C^s as soon as possible after the onset of the disease in the LEC. 

A connectopathy like AD is highly complex; therefore, countless approaches have been and are currently being made to understand and arrest it. Here, we describe one such approach using a strongly positively charged polypeptide that selectively targets AβOs as potentially therapeutic. Chakravarthy et al. [72] have reported a cationic amyloid-β binding peptide (ABP) that selectively binds to high molecular weight AβOs, *but poorly to physiologically functional monomeric Aβ_1–42_.* [73]. They have shown that this peptide targets AβO aggregates when microinjected into the hippocampi of *living* double-transgenic AD mice harboring PSEN1dE9 and APP_SWE_ transgenes [73] and also ex vivo in brain sections of both transgenic mice and postmortem human AD patients. Interestingly, ABP, which can also inhibit membrane-associated PKC activity) [75,76] is actually the 1276–1314 region of the human PCM1 described above. 

Recently a bifunctional fusion protein has been generated in which a blood-brain barrier crossing single-domain antibody FC5 is fused to an amyloid binding peptide (ABP) via mouse IgG2a Fc fragment (FC5-mFc2a-ABP). In aged transgenic McGill-R-Thy1-APP rats expressing human APP_751_ with familial AD mutations, 5-week treatment with KG207-M markedly reduced brain Aβ levels measured by positron emission tomography reversed hippocampal atrophy and improved resting state functional connectivity [432].

It also appears that the pathology’s advance through the cortex is sustained by signals from AβO^−^s → Ca^2+^•CaSR to make and secrete more AβOs [78,113,354]. Therefore, any drug that can inhibit Ca^2+^•CaSR signaling could collaborate with an AβOs binder to stop the spread of the connectopathy (Figure 1). Such a family of drugs, specifically the CaSR-inhibiting ‘calcilytics’ (e.g., NPS2143), have recently been shown to inhibit the secretion of Aβ and *all of their toxic actions* in cultured human astrocytes and neurons [78,113,354]. Moreover, these drugs have been pre-clinically and clinically shown to be well-tolerated by rodents and humans [45,356,433,434]. Thus, for example, delivering a double therapeutic consisting of an AβOs binder and a safe catalytic like NPS 2143 before MCI or even later might stop the further spreading of LOAD/SAD, likely without reversing already inflicted damage. However, unless we can stop aging or otherwise eliminate the old brain’s AβOs producing machinery, it will resume hyper-accumulating Aβ_x–42_s and seeding AβOs cocktails if we should stop treatment. Therefore, such a combined therapy would likely need to be given intermittently for life.

## Abbreviations

AβPP	Amyloid-β pecursor protein
Aβ	Amyloid-β
AβOs	Amyloid-β oligomers
AD	Alzheimer’s disease
α-7nAChRs	Nicotonic acetylcholine receptors
AMPA	α-amino-3-hydroxy-5-methyl-4-isoxazolepropionic acid
ANTs	Astrocyte•Neuron Teams
Apo E	Apolipoprotein-E
BACE1	Beta-site amyloid precursor protein cleaving enzyme-1 (β-secretase 1)
BBB	Blood–Brain Barrier
BDNF	Brain-derived neurotrophic factor
BOLD fMRI	Blood Oxygen Level Dependent functional Magnetic Resonance Imaging
CaSRs	Ca^2+^-sensing receptors
Cdk	Cyclin-dependent kinase
CSF	Cerebrospinal fluid
DG	Dentate gyrus
DMN	default-mode network
EAATs	Excitatory Amino Acid Transporters
EOAD/FAD	Early-Onset or Familial AD
EC	Entorhinal cortical
ECM	Extracellular Matrix
EVs	Extracellular Vesicles
GSK3β	Glycogen synthase kinase-3 β
IDPs	Intrinsically disordered proteins
ISF	Interstitial fluid
LEC	Lateral entorhinal cortex
LRP1	LDL receptor-related protein 1
MAPK (ERK)	Mitogen-activated protein kinase (extracellular signal-regulated kinase)
MARCKS	Myristoylated alanine-rich C kinase substrate
MCI	Mild Cognitive Impairment
NAHAs	Normal Adult Human cerebral Astrocytes
NFTs	Neurofibrillary tangles
NMDARs	N-methyl-D-aspartate receptors
PCM1	pericentriolar material-1
PET	Positron Emission Tomography
PKC	Protein Kinase C
PrP^C^	Cellular prion protein
PQCs	protein quality and quantity control systems
ROS	Reactive Oxygen Species
LOAD/SAD	Late onset or sporadic AD
HPTOs	Hyper-phosphorylated tau oligomers
PQCs	Protein quality and quantity control systems
PSD	Postsynaptic density
SDC	somatodendritic compartment
